# A stable explant culture of HER2/neu invasive carcinoma supported by alpha-Smooth Muscle Actin expressing stromal cells to evaluate therapeutic agents

**DOI:** 10.1186/1471-2407-8-119

**Published:** 2008-04-24

**Authors:** Marie P Piechocki

**Affiliations:** 1 Department of Breast Cancer Immunotherapy, Wayne State University and Karmanos Cancer Center, Detroit, MI, USA

## Abstract

**Background:**

To gain a better understanding of the effects of therapeutic agents on the tumor microenvironment in invasive cancers, we developed a co-culture model from an invasive lobular carcinoma. Tumor cells expressing HER2/neu organize in nests surrounded by alpha-Smooth Muscle Actin (α-SMA) expressing tumor stroma to resemble the morphology of an invading tumor. This co-culture, Mammary Adenocarcinoma Model (MAM-1) maintains a 1:1 ratio of HER2/neu positive tumor cells to α-SMA-reactive stromal cells and renews this configuration for over 20 passages in vitro.

**Methods:**

We characterized the cellular elements of the MAM-1 model by microarray analysis, and immunocytochemistry. We developed flow cytometric assays to evaluate the relative responses of the tumor and stroma to the tyrosine kinase inhibitor, Iressa.

**Results:**

The MAM-1 gene expression profile contains clusters that represent the ErbB-2 breast cancer signature and stroma-specific clusters associated with invasive breast cancers. The stability of this model and the ability to antigenically label the tumor and stromal fractions allowed us to determine the specificity of Iressa, a receptor tyrosine kinase inhibitor, for targeting the tumor cell population. Treatment resulted in a selective dose-dependent reduction in phospho-pMEK1/2 and pp44/42MAPK in tumor cells. Within 24 h the tumor cell fraction was reduced 1.9-fold while the stromal cell fraction increased >3-fold, consistent with specific reductions in phospho-pp44/42 MAPK, MEK1/2 and PCNA in tumor cells and reciprocal increases in the stromal cells. Erosion of the tumor cell nests and augmented growth of the stromal cells resembled a fibrotic response.

**Conclusion:**

This model demonstrates the specificity of Iressa for HER2/neu expressing tumor cells versus the tumor associated myofibroblasts and is appropriate for delineating effects of therapy on signal transduction in the breast tumor microenvironment and improving strategies that can dually or differentially target the tumor and stromal elements in the microenvironment.

## Background

The development of targeted therapies for the specific inactivation of receptor tyrosine kinase oncogenes involved in tumor initiation and progression has lead to the ability to target signal transduction as a modality for cancer treatment and prevention [1,2]. ZD1839 (gefitinib, Iressa^®^), an orally active, selective EGFR-Tyrosine Kinase Inhibitor (TKI) that blocks signal transduction pathways implicated in proliferation and survival of cancer cells and other host-dependent processes that promote cancer growth [3,4]. To date, we have already demonstrated the efficacy of Iressa against mammary and salivary gland tumor cell lines derived from transgenic mice that over-express the activated rat HER2/neu [5,6]. These studies focused mainly on the direct effects of Iressa on tumor cells. More recently, we have determined that Iressa can also prevent the outgrowth and progression of mammary and salivary gland cancers from early hyperplasias [7]. During these studies, we observed significant changes in the microenvironment as a result of treatment. It has been widely recognized that the tumor microenvironment plays a major role in dictating tumor behavior and progression as well as response to therapy. To better define, characterize and understand the effects of Iressa on the tumor and its microenvironment we developed a stable model of HER2/neu positive mammary tumor cells in co-culture with alpha-Smooth Muscle Actin (α-SMA)-positive stromal cells that recapitulate the microenvironment of an invasive carcinoma.

Several organotypic breast cancer models and co-cultures have been described. These include admixtures of tumor cells and fibroblasts or stromal cells [8-10], mammary tumor cells grown as spheroids [11] or 3-dimensional scaffolds [12], organ cultures [13] and orientated lumen forming acinar cultures [14,15]. These can be laborious to maintain and difficult to analyze without specialized reagents and equipment. We have developed a self-renewing model that circumvents some of these technical barriers and has proven to be stable, reliable and user friendly. We have identified several advantages to the Mammary Adenocarcinoma Model (MAM-1) for screening preventive and therapeutic agents, emphasizing the need to evaluate therapies in the context of homotypic microenvironment.

MAM-1 is immortal and faithfully recapitulates the morphology of invasive carcinomas that arise in BALB-NeuT transgenic mice, a model for HER2/neu driven lobular carcinoma [16]. MAM-1 grows rapidly in vitro and in vivo and maintains a 1:1 tumor-to-stroma ratio with routine passaging. This ratio can be manipulated with differential trypsinizations. This configuration is stable for over 20 passages. In MAM-1 there is no need for separate cultures, special media or culture conditions. MAM-1 can be used to test any agent or type of therapy, especially HER2 and stroma targeted therapies including biological and immunotherapies. Using MAM-1 treatment effects can be followed by out growth assays in vitro and in vivo (residual tumorigenic potential) in BALB/c mice. A key advantage to MAM-1 is the ability to simultaneously evaluate tumor cells and stromal cells using convenient markers (i.e., α-SMA, HER2) that are stable and suitable for flow cytometry (FACs) and immunofluorescent imaging. Furthermore, cells can be fractioned, based on these stable markers, to generate lysates for IP, Western blot, and multiplex bead arrays or generate RNA and DNA for microarray and methylation analyses. Finally, MAM-1 is suitable for use in assays that evaluate invasive and angiogenic potential of cells.

In this paper we describe the development of the MAM-1 co-culture model and methods for manipulating and analyzing it to evaluate mechanism(s) of the receptor tyrosine kinase inhibitor, Iressa. We further resolve a dynamic reciprocity between tumor and stromal cell populations during growth and treatment.

## Methods

### BALB-NeuT Transgenic animals

Two stock BALB-NeuT transgenic males were obtained through collaboration with Dr. Guido Forni. The BALB-NeuT strain originated from a transgenic CD1 random-bred breeder male mouse (no. 1330) carrying the mutated rat HER2/*neu *oncogene driven by the MMTV promoter [16]. The mutated gene encodes a single point mutation that replaces the valine residue at position 664 in the transmembrane (TM) domain of p185/neu with glutamic acid. This mutation promotes p185/neu homo- and heterodimerization and transforms the HER2/*neu *protooncogene into a dominant transforming oncogene. In these studies animals were used for tissue harvest only. Nonetheless, all animal research was conducted following an approved protocol filed with the Animal Investigation Committee at Wayne State University that oversees the Division of Laboratory Animal Resources (DLAR) at this institution in strict accordance with NIH guidelines.

### Iressa™ and cell lines

"Iressa™" (Zeneca Pharmaceuticals Macclesfield, Cheshire) ZD1839, 4-(3-chloro-4-fluoroanilino)-7-methoxy-6-(3-morpholinopropoxy) quinazoline was suspended in DMSO at 10 mM and stored at -20°C until use. Dilutions were prepared in culture medium to make final concentrations ranging from 0.25–10 μM. The Bam1a cell line has been previously described and characterized [6].

### Establishment and Maintenance of the MAM-1 co-culture Model

The MAM-1 co-culture model was established from a BALB-NeuT transgenic mouse with an advanced mammary gland lobular carcinoma involved with hemorrhage. The tissue was removed asceptically and rinsed extensively in sterile PBS. The tissue was minced and dissociated briefly with collagenase. Cells were washed extensively with complete medium and explanted into tissue culture media. Cultures grew in an organization of tumor cell nests surrounded by myofibroblasts and maintained a consistent 1:1 ratio of tumor cells to stromal cells. These cultures were maintained *in vitro*in Dulbecco's modified Eagle's medium (DMEM) with high glucose and supplemented with 10% heat inactivated Serum Supreme (BioWhittaker, St. Louis, MO), 0.5 mM sodium pyruvate, 2 mM L-glutamate, 0.1 mM MEM nonessential amino acids, 100 units/ml penicillin and 100 μg/ml streptomycin and 10 μM Dexamethasone. Inclusion of dexamethasone, impairs the growth of contaminating fibroblasts and endothelial cells with limited proliferative and immortalization potential. Cells were cultured in 10% CO_2_, media was changed every 3–4 days until confluence and split at a 1:5 ratio for routine passaging. -Co-cultures can be expected to maintain a 1:1 ratio during active growth. This ratio may be altered if cultures are kept beyond confluence when stromal cells continue to grow. At time of passage, the majority of stromal cells are collected separately by a brief trypsinization. The remaining co-culture is further trypsinized and monodispersed and collected separately. Both collections are counted by hemacytometer which readily allows for distinguishing between the tumor (small) and stromal (large) cells. If need be, the cell ratios can be readjusted to accommodate the desired 1:1 ratio. All *in vitro *data presented in this manuscript used MAM-1 between passages 5–20.

### Immunohistochemistry

At time of necropsy, tumor tissues were fixed in 10% neutral buffered formalin and paraffin embedded using standard histochemical techniques. Blocks were sectioned 4-micron. Tissue sections were stained with Hematoxylin and Eosin for basic histological evaluation. For immunodetection of tissue antigens, histological grade primary antibodies were applied to the samples and incubated according to manufacturer's recommendation: HER2, PAD: Z4881, cat# 08-1204 2^nd ^Gen; PCNA, cat# 08-1110 (Zymed, South San Francisco, CA), PCNA Ab-1 clone (PC10) cat# MS-106-R7; Actin, Smooth Ab-1 (1A4) cat# MS-113-R7 (Neomarkers, Fremont, CA). Samples were washed and labeled using the SuperPicTure™ Polymer Detection Kit, cat#87-9263; Zymed), developed with DAB Substrate and counterstained with hematoxylin. Samples were evaluated using a Zeiss microscope and images were collected through a Sony 970 CCD camera interfaced with the MCID5+ imaging software package. Alternatively images were collected using a Nikon inverted microscope equipped with a SPOT digital cooled camera and imaging software. Stained sections were evaluated by a board certified pathologist to generate descriptions.

### Flow cytometric analysis of MAM-1 co-cultures

A variety of antibodies were used for flow cytometric analyses. Selection of antibody combinations was based on fixation, host species, avidity/affinity for specific epitopes and antigen density. Antibodies used in these studies included: antibody to the rat Her2/neu (Ab-9, clone B10, cat# MS-326); erbB-2 (Ab-15, clone 3B5, cat# MS-599, NeoMarkers, Fremont, CA). CD24 (M1/69) sc-19651-PE; CD29 (Integrin β1 (HMβ1-1) sc-19656-PE; p-c-Jun (Ser63)-PE (KM-1) sc-822-PE all from Santa Cruz Biotechnology, Santa Cruz, CA. Ready to use histological grade antibodies that were also used on formaldehyde fixed samples prepped for flow cytometry or immunofluorescence included: (HER2, PAD: Z4881, cat# 08-1204 2^nd ^Gen; PCNA, cat# 08-1110 Zymed, South San Francisco, CA). or PCNA Ab-1 clone (PC10) cat# MS-106-R7; Actin, Smooth Ab-1 (1A4) cat# MS-113-R7 or cat# RB-9010-R7, Neomarkers, Fremont, CA. Phospho-specific antibodies from Cell Signaling Technologies, (Beverly, MA) used in FACS and Immunofluorescence included: p-p44/42 MAP kinase (Thr202/Tyr204, #9101), p-MEK ½ (Ser217/221. #9121)

Routine cell surface staining of fresh cell cultures was as previously described [5]. For evaluation of intracellular antigens, MAM-1 were plated in 6 well tissue cultures plates to produce a confluent, organized co-culture (1:1 tumor to stroma cell ratio) within 2–3 days of seeding. Cells were harvested at specific time points after treatment with trypsin/EDTA, quenched with complete medium and collected by centrifugation. Pellets were resuspended in 1 mL of PBS and monodispersed by passing through a small bore pipet. Cells were fixed by titrating in 5% Formaldehyde to a final concentration of 1%, incubated at 37°C for 10 minutes, then chilled on ice for 2 minutes. Absolute, ice-cold (-20°C) methanol was added while vortexing to a final concentration of 90%. Samples were vortexed rigorously and incubated on ice for 30 minutes. Cells were collected by centrifugation and blocked for 30 minutes on ice with chilled FACscan buffer (PBS supplemented with 2% serum and 0.1% sodium azide). Cells were washed once with fresh FACscan buffer prior to the addition of primary antibodies or isotype controls and stained overnight at 4°C in accordance to the manufacture's guidelines. Cells were washed twice and stained with the appropriate -PE or -FITC conjugated secondary antibodies: goat anti-mouse IgG-PE, cat# 111-116-144; donkey anti-rabbit IgG-FITC, cat#711-096-152; donkey anti-mouse IgG-PE, cat# 715-116-150 (Jackson ImmunoResearch, West Grove, PA) at 1:50 to 1:200 on ice for 45 min. After staining, samples were washed twice with FACscan buffer, placed on ice and evaluated by flow cytometry using the dual-color laser option (FL1 v. FL2) in the FACsCaliber. At least 20,000 events were collected for every sample. Data were analyzed using WinMDI version 2.8 software. Importantly, all experiments were reproduced at least three times using MAM-1 of different passage numbers and overlapping experimental treatments and time points. The specificities of all stains were validated by using various combinations of monoclonal and polyclonal antibodies for each antigen and different secondary labels. All phospho-specific markers and PCNA stains were evaluated by direct reciprocal dual-staining for the test antigen with in combination with antibodies to ErbB2 and α-SMA to verify that all ErbB-2 negative responses were identical to α-SMA positive responses and vice versa.

### Immunofluorescence

MAM-1 suspensions were plated on glass cover slips in 6 well plates in complete media and grown to 90–95% of confluence and treated as described in the text. Following treatment, cells were fixed and permeabilized by immersion in ice-cold 100% methanol and incubated at -20°C for 10–20 min. Methanol was aspirated and cover slips were air-dried, washed and blocked prior to administration of primary antibodies. Primary antibodies were diluted and incubated on cover slips according to the recommendations for each specific antibody. Cover slips were washed 4 times before adding the secondary FITC- or TRITC-conjugated goat anti-mouse or rabbit IgG (cat# 115-096-071, 111-096-046, 111-116-144 or 115-116-071; Jackson ImmunoResearch, West Grove, PA) antibodies diluted 1:50-1:200 in FACscan buffer. Samples were incubated with secondary for 45 min. at room temperature in the dark. Stained cover slips were washed and mounted in Fluorescent mounting media (cat# HT08, Oncogene Science) containing DAPI for nuclear counterstaining. Slides were visualized at 100× under oil with a Zeiss microscope equipped with a Sony 970 digital cooled camera. Fluorescence photomicrographs were imaged using MCID5+ software. Live cell images and phase contrast of cells fixed in 6-well culture dishes were evaluated with a Nikon inverted microscope and imaged using the attached SPOT digital cooled camera and imaging software.

### Gene Expression Analysis

Total RNA was isolated from triplicate wells of Bam1a cells [6] or MAM-1 co-cultures that were treated for 24 h with complete media containing 0 or 1 μM Iressa. Total RNA was isolated using RNeasy kit (Qiagen, Carlsbad, CA) and sent to SuperArray Bioscience Corporation (Frederick, MD) and processed utilizing the GEArray Hybridization and Analysis Service and Agilent Mouse Genome CGH Microarray 44A that has *43,000+ *annotated gene sequences with well-characterized genes represented by 1+ probes and cancer-relevant genes by 2+ probes. In some comparisons Universal mouse RNA provided by Agilent was also used in the analyses for normalization. Three independent RNA preparations per condition were analyzed on three separate chips and used to generate the analysis report. To generate lists of highly expressed genes, RNA from MAM-1 was labeled in red color (cy5), universal RNA was labeled in green (cy3). 1) In **treated vs **control comparison, the treated is labeled in red color (cy5), control is labeled in green color (cy3). Fold change (**treated/**control) = Normalized ratio value (cy5/cy3); fold change cutoff >2 fold (up regulate) or < 1/2 (down regulate); confidence cutoff >0.95.

## Results and Discussion

### Histological analysis of the primary tumor tissue used to derive MAM-1

**Figure 1:**
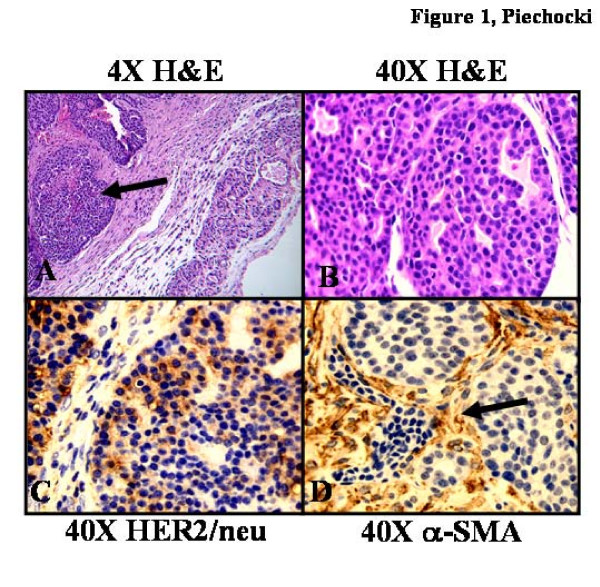
**Histological analysis of the primary tumor tissue used to derive MAM-1**. The tumor was obtained from the #8 mammary gland of a BALB-NeuT transgenic mouse. A. Hematoxylin and Eosin staining revealed a prominent fibrovascular response associated with the tumor (4×). The tumor forms a discrete nodule within a thin rim of collagen (pseudocapsule). The tumor is made of epithelial cells, arranged in ill-defined nests (*arrow*) that are separated by fine fibrous septa. Discrete acini, delimiting central lumens, and filled with lightly eosinophilic secretions, confer to these epithelial nests a distinct overall, sieve-like (cribriform) configuration. Necrotic material and focal hemorrhage accumulates within the tumor, in a punctate fashion (comedo-type necrosis). B. Tumor cells show homogenous cytological features and only mild pleomorphism. They are polygonal, with a relatively low N/C ratio, eosinophilic cytoplasm, round central nuclei with finely dispersed chromatin and infrequent macronucleoli and undergo frequent mitoses (40×). C. Immunohistochemical analysis of HER2/neu shows strong staining in the majority of the tumor cells, in a cytoplasmic and membranous distribution. D. Immunohistochemical analysis for α-SMA detected strong cytoplasmic staining associated exclusively with the stromal cells (*arrow*) surrounding the tumor cell nests. The antigens were detected by DAB (brown) and nuclei counterstained with hematoxylin (blue). Images were photographed under 40× objective.

The tumor was obtained from a BALB-NeuT transgenic mouse bearing a lesion that involved a hemorrhage of the #8 thoracic mammary gland. The pathology report describes the tumor as forming a discrete nodule, most of which is contained within a thin rim of collagen (pseudocapsule) with rare isolated acini penetrating it (Fig. 1A). The tumor is made of epithelial cells, arranged in ill-defined nests that are separated by fine fibrous septa. Discrete acini, delimiting central lumens, and filled with lightly eosinophilic secretions, confer to these epithelial nests a distinct overall, sieve-like (cribriform) configuration. Necrotic material and focally hemorrhage, frequently accumulates within the tumor, in a punctate fashion (comedo-type necrosis). Tumor cells show homogenous cytological features and only mild pleomorphism (Fig. 1B). They are polygonal, with a relatively low N/C ratio, eosinophilic cytoplasm, round central nuclei with finely dispersed chromatin and infrequent macronucleoli and undergo frequent mitoses. This description is consistent with the morphology of the lobular carcinomas that arise in BALB-NeuT transgenic mice [16].

The sample of this lesion that was explanted and processed for growth in vitro, demonstrates a significant stromal cell component (Fig. 1). By immunohistochemistry we observed that HER2/neu staining was strong and present in the majority of the tumor cells (Fig. 1C), in a cytoplasmic and membranous distribution and that α-SMA staining was strong and exclusively expressed in the cytoplasm of the stromal cells that were involved in the fibrous sheaths surrounding the tumor cell nests (Fig. 1D).

### Growth and maintenance of MAM-1 explant cultures

In mice transgenic for the activated rat HER2/neu under the MMTV promoter, expression of the oncogene in the mammary gland epithelium gives rise to an alveolar type of lobular carcinoma that requires an angiogenic switch for tumor onset and progression to invasive cancer [16]. Coordinated epithelial-stromal interactions that are required for mammary morphogenesis and development are also critical for tumor progression in this model. In the case of human breast cancers, stromal alterations are also integral to the evolution and progression of breast cancer [17,18].

**Figure 2:**
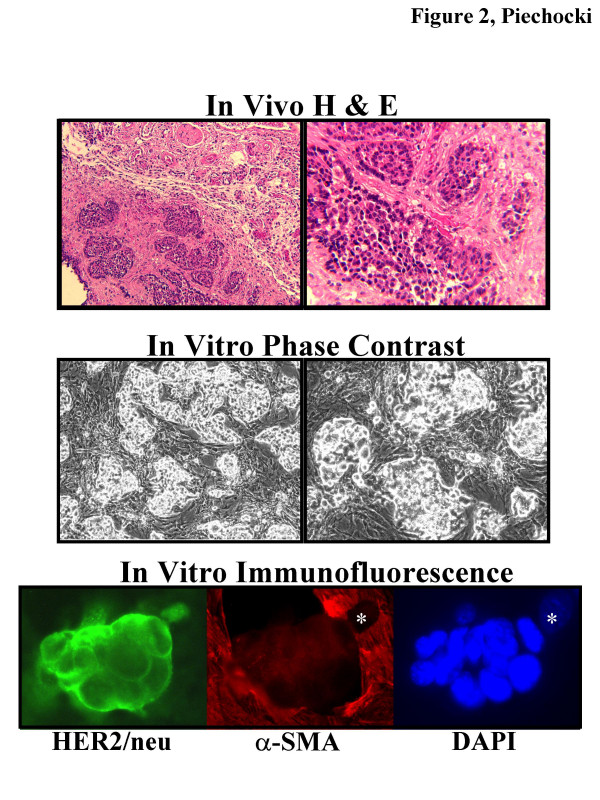
**Comparison of Tumor tissue morphology in vivo with Explants grown in vitro**. In Vivo H&E (Top panel) is Hematoxylin and Eosin staining of the primary tumor lesion used for generating explant cultures and shows tumor cell nests in a fibrofatty matrix within the breast stroma taken with a 10× objective (left) and 25× objective (right). In Vitro Phase Contrast photomicrographs (Middle Panel) depict the growth and morphology of MAM-1 explant cultures in vitro. Photomicrographs were taken with a (10×, left) or (20×, right) objective. Note in particular the morphological similarities between the explants grown in vitro and resemblance to the tumor in vivo. In Vitro Immunofluorescence (Bottom Panel) of MAM-1 grown on coverslips and dual-stained for HER2/neu (FITC, green) and α-SMA (TRITC, red) and counterstained for DAPI depicting the nuclei in blue. HER2/neu staining is strong and specific for tumor cells while α-SMA staining is exclusively associated with the cells surrounding the tumor cell nests and localized to the actin-based cytoskeleton. Asterisk depicts nucleus of stromal cell for orientation.

Preservation of the breast cancer microenvironment is critical for evaluating therapeutic agents especially when designing modalities that target invasive disease. Thus, we sought to establish a homotypic system representing invasive breast cancer by explanting the mammary tumor and associated stromal cells to develop a co-culture model. Upon explantation from the tumor, primary cultures that grew in vitro produced a co-culture that organized as multi-cell layered nests of tumor cells surrounded by concentric rings of stromal cells. These arrangements bear a striking similarity to the morphology of the primary tumor lesion (Fig. 2). This configuration was stable and was able to renew itself under limiting cell dilutions, these co-cultures were designated as MAM-1.

Passage of MAM-1 stock cultures at a 1:5 ratio maintained this organized growth pattern to reproducibly generate co-cultures that consisted of 50% tumor cells and 50% stromal cells. Cultures maintained in this way achieved 95% confluence within 5–7 days reproducibly for out to 20 passages. There was a tendency for stromal cells to proliferate more rapidly. Excess stromal cell growth was controlled by a mild trypsinization that released the superficial layer of stromal cells from the culture. At days 3–5 post-confluence, tumor cell nests formed spheroids that eventually pinched off and were capable of re-establishing the MAM-1 configuration when plated on fresh tissue culture plastic. Thus, spheroid formation is a natural progression in this co-culture model and does not require the elaborate procedures or growth conditions described for other model systems [8,11,12]. MAM-1 is also capable of growth in soft agar. However, if not in association with MAM-1 tumor cells, stromal cells will tend to form monolayers on top of or beneath the agar. One reason for the faithful renewal of this self-contained co-culture model can be attributed to the properties of syngeneic tumor associated stromal cells which have a strong influence on mammary tumor cell growth and gene expression [8,11,18]. Further studies are underway, to establish the genetic relationship between the cells of the stroma and tumor (i.e. karyotype analysis) and to determine the potential of each cell type to develop tumors in vivo.

### Validation of tumor specific and stroma specific antigens in explant cultures by Immunofluorescence and Flow cytometry

As suggested by immunohistochemical analysis of the primary tumor (Fig. 1) we expected the tumor cells in these co-cultures to express HER2/neu and the stromal cells to express α-SMA. We observed that expression of HER2/neu was exclusive to the tumor cell nests with a high level of distribution in the membrane and cytoplasm (Fig. 2). HER2/neu expression was at background level in the stromal cells, and α-SMA was strongly expressed in the cytoplasmic actin-based microfilaments exclusively in these cells. In this co-culture setting, strong and consistent expression of both antigens, tumor-associated HER2/neu and stromal-associated α-SMA were stable out to 20 passages. Using these two antigens as markers to identify and separate tumor from stroma cells, we adapted our staining method for flow cytometric analysis that would enable us to evaluate responses in each cell type.

**Figure 3:**
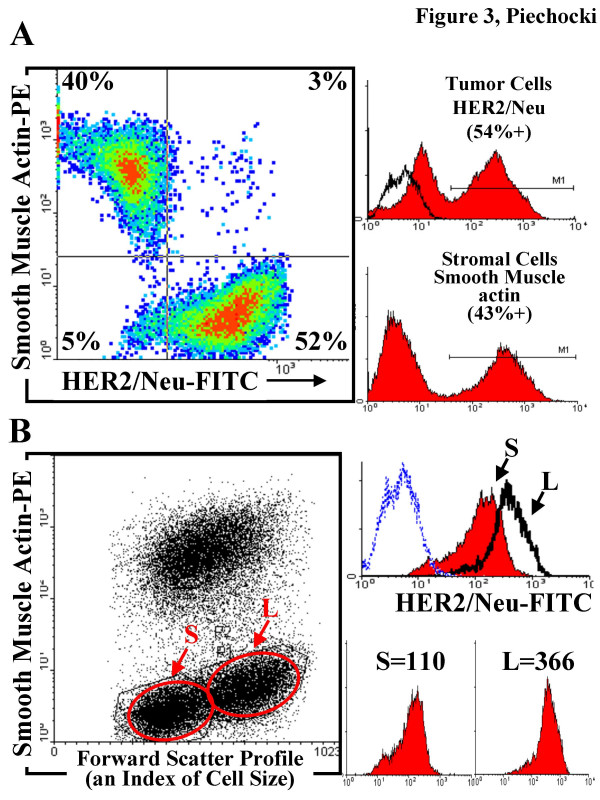
**Flow Cytometric Analysis of MAM-1 co-cultures. A. Density plot of MAM-1 co-culture stained for HER2/neu and α-SMA**. MAM-1 co-cultures were stained with the same ready-to-use, histological grade antibodies that were used for IHC and IC to detect HER2, PAD: Z4881 and α-SMA, (1A4) in tissues. Primary antibodies were labeled with -PE and FITC conjugated secondaries as described in the methods. Quadrant analysis revealed that MAM-1 cultures consisted of 52% HER2/neu^+^, α-SMA^- ^tumor cells, 40% HER2/neu^-^, α-SMA^+ ^stromal cells, a 5% population of HER2/neu^-^, α-SMA^- ^cells and a 3% population of HER2/neu^+^, α-SMA^+ ^cells which may represent cells undergoing epithelial-mesenchymal transition. At the right, Histogram analysis of the separate FL1 (top) and FL2 (bottom) events indicates 54% of the culture as positive for HER2/neu (top) and 43% of cells as positive for α-SMA (bottom) which is consistent with the quadrant analysis. Filled peaks are specific antibody stained cells and open peaks represent background staining with the isotype control. B. Subpopulation analysis of Tumor cell fractions in MAM-1 co-cultures based on Forward scatter profiles. Dot plot analysis of a-SMA-PE stained cells versus Forward scatter indicates the presence of two distinct subpopulations of different size in the a-SMA negative fraction labeled as S (small) and L (large). Histogram analysis of these gated subpopulations (defined by the circles) for HER2/neu specific staining indicates that the small population has lower levels of HER2/neu, a mean channel fluorescence of 110 compared to the large population enriched in dividing cells, with a mean channel fluorescence of 366.

As shown in Fig. 3A, the MAM-1 co-culture is readily fractionated into a HER2/neu+, α-SMA-tumor subpopulation that accounts for 50–55% of the culture, a HER2-, α-SMA+ stromal cell population that accounts for 40–45% of the culture, a HER2/neu+, α-SMA+ population which may represent a stem cell or cells undergoing mesenchymal transition and typically accounts for 3–5% of the culture and finally a double negative population that appears to represent a fibroblast population that typically accounts for 3–5% of the culture.

Whether or not a "true" mammary stem cell exists in this co-culture is under investigation. A variety of candidate mammary stem cell markers are expressed by the different subpopulations. In mature MAM-1 co-cultures we have determined that the tumor cell population is HER2/neu^+^, CD24^lo/med^, CD29^hi^, SMA^- ^and the stromal population is HER2/neu^-^, CD24^neg^, CD29^hi^, SMA^+^. Since CD24 negative, lo and hi populations correspond to nonepithelial, basal/myoepithelial and luminal epithelial cells respective [19], we consider our "stromal" cells to be nonepithelial and our "tumor" cells to be a mixture of luminal and basal/myoepithelial phenotypes. In so much as CD24^+^CD29^hi ^phenotype is enriched in its ability to reconstitute the essential elements of the mammary gland, a mammary tumor stem cell-like subpopulation may be present in these MAM-1 cultures. Since the "oncogenic" activated rat HER2/neu genetic lesion is expressed in the mammary stem cell of the donor BALB-NeuT transgenic mice, it is possible that this co-culture model may support a limited degree of cell differentiation from a stem-like progenitor that is in the HER2/neu positive subpopulation. Further support for a stem cell component in this self-renewing co-culture model is provided by the high level of CD44 in MAM-1 and enriched expression of vimentin and cytokeratin 19 in MAM-1 compared to the cloned Bam1a cell line described later.

**Figure 4:**
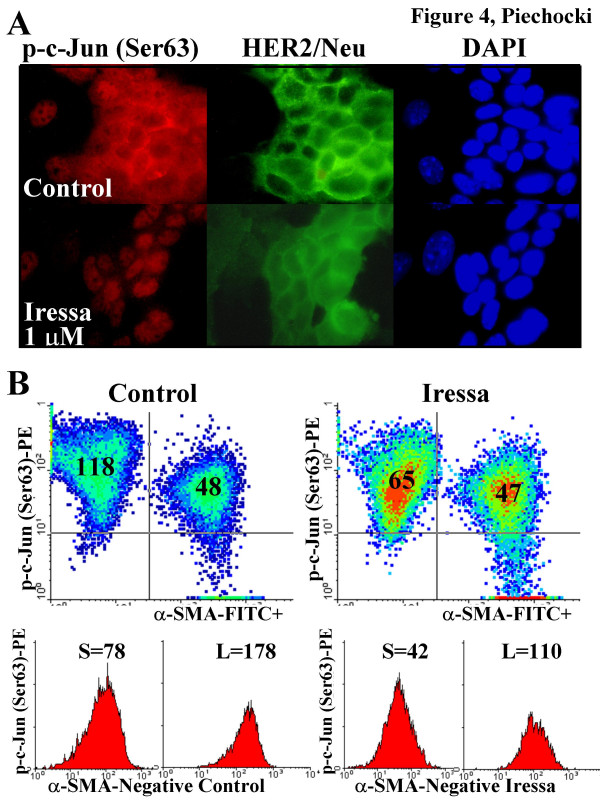
**Evaluation of p-c-Jun (Ser63) levels and subcellular distribution in MAM-1 co-cultures treated with Iressa by A. Immunofluorescence and B. Flow Cytometry**. MAM-1 were subcultured on coverslips or in 6-well plates and grown to ~95% confluence and then treated by replacing the conditioned media with fresh media that contained diluent (.001% DMSO, Control) or 1 μM Iressa for 1 hour prior to fixing and evaluation as described in the methods. A. Immunofluorescent photomicrographs were taken with the 100× objective under oil immersion of cells that were double labeled for p-c-Jun (Ser63) in red (TRITC) and HER2/neu in green (FITC) and counterstained with DAPI (blue) to define the nuclei. B. Flow cytometric analysis of control (left) and Iressa-treated (right) MAM-1 cultures. Cells were dual labeled for p-c-Jun (Ser63) and α-SMA. Density plots compare p-c-Jun (Ser63) expression levels in the α-SMA negative and positive subpopulations in control and Iressa-treated MAM-1 cultures. Mean Channel fluorescent values for p-c-Jun (Ser63) are indicated in the respective quadrants. Histogram analyses for p-c-Jun (Ser63) expression in the α-SMA negative subpopulations were generated from dot plots of p-Jun (Ser63)-PE versus Forward Scatter and gating on the respective the S (small) and L (large) fractions. Histograms of these tumor cell subpopulations demonstrate different baseline levels (Left) of p-c-Jun (Ser63) levels and Iressa responsiveness (Right) in small (S) and large (L) cells. Mean channel fluorescent values for p-c-Jun (Ser63) levels are indicated above the peaks. Parallel samples were dual stained for HER2/neu and p-c-Jun (Ser63) to verify these data (not shown).

Using flow cytometric analysis we were able to further subfractionate the HER2/neu (tumor) population based on their Forward Scatter Profile, which is an index of cell size. These different size tumor cell subpopulations may represent cells enriched at different phases of the cell cycle, smaller cells tend to be enriched for cells in G0 and larger cells tend to be enriched for cells in G2/M (not shown). We typically observe an equal distribution of small (S) and large (L) cell subpopulations in the mammary tumor cell fraction (Fig. 3B). We observed that the larger cell population (L) had approximately >3-fold higher level of HER2/neu expression on the cell surface. If this subpopulation represents a high proportion of cells in G2/M antigens and drug responses that are differentially sensitive to the phase of cell cycle can be detected/identified in the different sized tumor subpopulations (by forward scatter) and then confirmed and correlated with further analysis of the cell cycle distribution. For example, MAM-1 co-cultures that are treated for 1 hour with Iressa show a redistribution of p-c-Jun (Ser63) to the nucleus (Fig. 4A) and HER2/neu to the cytosol. In addition to redistribution from the cytosol, there was an overall decrease tumor cell associated phospho-c-Jun (i.e., in the α-SMA negative cells) but not in the α-SMA positive stromal cells (Fig. 4B). The observed 45% decrease in overall tumor cell phospho-c-Jun resulted from a 46% decrease that corresponded to the small cells and a 38% decrease in the large cells. Thus, small, non-dividing cells are about 20% more responsive to Iressa in terms of c-Jun phosphorylation. This further implies that large, dividing cells, which are enriched in HER2/neu receptors (Fig. 3B) and have a higher baseline level of phospho-c-Jun also have transient resistance to Iressa at this stage of the cell cycle. It has been widely documented that dividing cells often have a higher level of intrinsic resistance to a variety of chemotherapeutic agents.

### The MAM-1 transcriptome has a genetic signature of ErbB-2 breast cancers and desmoplasia observed in invasive breast cancers

We defined the MAM-1 transcriptome of highly expressed genes by comparing total RNA from a MAM-1 co-culture to a commercial preparation of universal mouse RNA which provides a balanced representation of normal and cancerous mouse tissues and cell lines. We used a cutoff value of >2-fold to be considered over-expressed and a p value cutoff > .05. A comprehensive listing of >2,000 known genes generated in this comparison is provided [see Additional file 1].

To identify genes associated with the tumor cell and stromal cell subpopulations in these cultures we compared the gene expression profiles of the Bam1a cell line, a cloned and characterized mammary carcinoma cell line developed from a BALB-NeuT mouse [6] and MAM-1 co-cultures. Bam1a was established from a soft agar colony and found to be immortal in vitro and tumorigenic in vivo and highly sensitive to Iressa and anti HER2/neu antibodies both in vitro and in vivo [6]. We identified clusters of genes that were highly expressed and common to Bam1a and MAM-1 co-cultures or differentially expressed between Bam1a and MAM-1. Table 1 is a representation of genes that are highly expressed by Bam1a and MAM-1 and part of the "ErbB-2 Signature" that is associated with many ErbB-2 expressing breast cancers [20-23]. In most instances the relative expression level of these signature genes was similar (within 2-fold) between Bam1a and MAM-1. This two-fold difference is likely to represent the dilution of tumor cell RNA with stromal cell RNA in the MAM-1 co-culture.

**Table 1:** Expression of HER2/neu ErbB-2 Breast Cancer Signature genes in MAM-1 Co-cultures and the Bam1a cell line

		**Expression Relative to Universal Mouse RNA^a^**		
**Agilent Accession**	**Genbank Accession**	**MAM**	**Bam1a**	**Gene Description and Symbol**
**Genes present in the tumor-specific HER-2/neu-Induced Gene Expression Signature described by Astolfi**				
A_51_P184886	NM_011920	5.27	19.10	Mus musculus ATP-binding cassette, sub-family G (WHITE), member 2 (Abcg2), mRNA [NM_011920]
A_52_P336142	NM_011920	4.90	17.77	Mus musculus ATP-binding cassette, sub-family G (WHITE), member 2 (Abcg2), mRNA [NM_011920]
A_51_P342567	NM_031185	9.10	6.60	Mus musculus A kinase (PRKA) anchor protein (gravin) 12 (Akap12), mRNA [NM_031185]
A_51_P445166	NM_019959	11.89	17.02	Mus musculus C1q and tumor necrosis factor related protein 1 (C1qtnf1), mRNA [NM_019959]
A_51_P455647	NM_009801	7.56	9.21	Mus musculus carbonic anhydrase 2 (Car2), mRNA [NM_009801]
A_51_P303160	NM_007482	14.32	23.23	Mus musculus arginase 1, liver (Arg1), mRNA [NM_007482]
A_52_P159232	NM_011926	20.60	24.52	Mus musculus CEA-related cell adhesion molecule 1 (Ceacam1), mRNA [NM_011926]
A_51_P183446	NM_001039185	10.69	12.16	Mus musculus CEA-related cell adhesion molecule 1, mRNA (cDNA clone MGC:18556 IMAGE:4218391), complete cds [BC016891]
A_51_P171728	NM_011926	9.77	12.34	Mus musculus CEA-related cell adhesion molecule 1 (Ceacam1), mRNA [NM_011926]
A_52_P240706	NM_007543	23.70	31.29	Mus musculus CEA-related cell adhesion molecule 2 (Ceacam2), mRNA [NM_007543]
A_51_P362066	NM_007695	8.34	3.89	Mus musculus chitinase 3-like 1 (Chi3l1), mRNA [NM_007695]
A_51_P495049	NM_009897	10.34	11.46	Mus musculus creatine kinase, mitochondrial 1, ubiquitous (Ckmt1), mRNA [NM_009897]
A_51_P343309	NM_009908	5.83	10.54	Mus musculus cytidine monophospho-N-acetylneuraminic acid synthetase (Cmas), mRNA [NM_009908]
A_52_P197963	NM_009936	7.33	10.23	Mus musculus procollagen, type IX, alpha 3 (Col9a3), mRNA [NM_009936]
A_52_P507877	NM_007729	41.31	30.23	Mus musculus procollagen, type XI, alpha 1 (Col11a1), mRNA [NM_007729]
A_51_P459477	NM_007729	34.68	28.45	Mus musculus procollagen, type XI, alpha 1 (Col11a1), mRNA [NM_007729]
A_51_P227280	NM_007786	53.04	56.89	Mus musculus casein kappa (Csnk), mRNA [NM_007786]
A_51_P227275	NM_007786	26.87	32.53	Mus musculus casein kappa (Csnk), mRNA [NM_007786]
A_52_P51078	NM_007801	23.49	35.47	Mus musculus cathepsin H (Ctsh), mRNA [NM_007801]
A_51_P275976	NM_010070		3.24	Mus musculus docking protein 1 (Dok1), mRNA [NM_010070]
A_52_P366842	NM_010070	2.49	4.94	Mus musculus docking protein 1 (Dok1), mRNA [NM_010070]
A_51_P158037	NM_013505	11.59	12.52	Mus musculus desmocollin 2 (Dsc2), mRNA [NM_013505]
A_52_P157158	NM_013505	42.42	46.62	Mus musculus desmocollin 2 (Dsc2), mRNA [NM_013505]
A_52_P252931	NM_013505	107.26	265.33	Mus musculus desmocollin 2 (Dsc2), mRNA [NM_013505]
A_51_P502614	NM_026268	4.28	6.46	Mus musculus dual specificity phosphatase 6 (Dusp6), mRNA [NM_026268]
A_51_P455866	NM_010125	27.80	29.60	Mus musculus E74-like factor 5 (Elf5), mRNA [NM_010125]
A_51_P307979	NM_007960		2.46	Mus musculus ets variant gene 1 (Etv1), mRNA [NM_007960]
A_51_P246166	NM_007969	10.06	10.23	Mus musculus extracellular proteinase inhibitor (Expi), mRNA [NM_007969]
A_52_P329207	NM_007969	13.14	12.84	Mus musculus extracellular proteinase inhibitor (Expi), mRNA [NM_007969]
A_51_P329394	NM_008034	6.64	8.48	Mus musculus folate receptor 1 (adult) (Folr1), mRNA [NM_008034]
A_52_P377791	NM_008557	30.34	40.63	Mus musculus FXYD domain-containing ion transport regulator 3 (Fxyd3), mRNA [NM_008557]
A_51_P282538	NM_008077	28.43	73.47	Mus musculus glutamic acid decarboxylase 1 (Gad1), mRNA [NM_008077]
A_52_P144310	NM_008077	27.33	68.46	Mus musculus glutamic acid decarboxylase 1 (Gad1), mRNA [NM_008077]
A_51_P486810	NM_030677	13.90	14.97	Mus musculus glutathione peroxidase 2 (Gpx2), mRNA [NM_030677]
A_51_P258409	NM_010423	13.98	30.78	Mus musculus hairy/enhancer-of-split related with YRPW motif 1 (Hey1), mRNA [NM_010423]
A_51_P365152	NM_010474	23.53	48.48	Mus musculus heparan sulfate (glucosamine) 3-O-sulfotransferase 1 (Hs3st1), mRNA [NM_010474]
A_52_P281702	NM_010518	2.12	3.08	Mus musculus insulin-like growth factor binding protein 5 (Igfbp5), mRNA [NM_010518]
A_51_P204153	NM_010518	4.25	10.70	Mus musculus insulin-like growth factor binding protein 5 (Igfbp5), mRNA [NM_010518]
A_51_P510156	NM_008491	9.56	10.11	Mus musculus lipocalin 2 (Lcn2), mRNA [NM_008491]
A_52_P153864	NM_010723	2.77	3.75	Mus musculus LIM domain only 4 (Lmo4), mRNA [NM_010723]
A_51_P394190	NM_010723	3.05	4.24	Mus musculus LIM domain only 4 (Lmo4), mRNA [NM_010723]
A_52_P415168	NM_019394	8.35	8.26	Mus musculus melanoma inhibitory activity 1 (Mia1), mRNA [NM_019394]
A_52_P415167	NM_019394	18.65	22.26	Mus musculus melanoma inhibitory activity 1 (Mia1), mRNA [NM_019394]
A_52_P174346	NM_019394	7.84	8.15	Mus musculus melanoma inhibitory activity 1 (Mia1), mRNA [NM_019394]
A_51_P220062	NM_008609	10.60	15.42	Mus musculus matrix metalloproteinase 15 (Mmp15), mRNA [NM_008609]
A_51_P139780	NM_009402	6.81	7.64	Mus musculus peptidoglycan recognition protein 1 (Pglyrp1), mRNA [NM_009402]
A_51_P195958	NM_009344	3.26	6.05	Mus musculus pleckstrin homology-like domain, family A, member 1 (Phlda1), mRNA [NM_009344]
A_52_P418791	NM_011254	15.11	16.38	Mus musculus retinol binding protein 1, cellular (Rbp1), mRNA [NM_011254]
A_51_P423484	NM_011254	8.26	7.45	Mus musculus retinol binding protein 1, cellular (Rbp1), mRNA [NM_011254]
A_52_P197215	NM_028777	2.76	4.29	Mus musculus SEC14-like 1 (S. cerevisiae) (Sec14l1), mRNA [NM_028777]
A_52_P197223	NM_028777	3.32	5.72	Mus musculus SEC14-like 1 (S. cerevisiae) (Sec14l1), mRNA [NM_028777]
A_51_P268094	NM_009255	22.21	38.32	Mus musculus serine (or cysteine) proteinase inhibitor, clade E, member 2 (Serpine2), mRNA [NM_009255]
A_52_P637988	NM_009199	40.88	62.00	Mus musculus solute carrier family 1 (neuronal/epithelial high affinity glutamate transporter, system Xag), member 1 (Slc1a1), mRNA [NM_009199]
A_51_P110759	NM_009199	20.06	26.16	Mus musculus solute carrier family 1 (neuronal/epithelial high affinity glutamate transporter, system Xag), member 1 (Slc1a1), mRNA [NM_009199]
A_52_P426605	NM_021301	23.10	30.57	Mus musculus solute carrier family 15 (H+/peptide transporter), member 2 (Slc15a2), mRNA [NM_021301]
A_51_P107362	NM_007706	13.23	14.79	Mus musculus suppressor of cytokine signaling 2 (Socs2), mRNA [NM_007706]
A_51_P346704	XM_128139	10.67	11.19	Mus musculus SRY-box containing gene 10, mRNA (cDNA clone MGC:32314 IMAGE:5027211), complete cds. [BC023356]
A_52_P192625	XM_128139	23.80	36.33	Mus musculus SRY-box containing gene 10, mRNA (cDNA clone MGC:32314 IMAGE:5027211), complete cds. [BC023356]
A_52_P161630	NM_145933		4.23	Mus musculus beta galactoside alpha 2,6 sialyltransferase 1 (St6gal1), mRNA [NM_145933]
A_51_P101375	NM_145933	2.35	5.29	Mus musculus beta galactoside alpha 2,6 sialyltransferase 1 (St6gal1), mRNA [NM_145933]
A_51_P183940	NM_016866	2.21	3.02	Mus musculus serine/threonine kinase 39, STE20/SPS1 homolog (yeast) (Stk39), mRNA [NM_016866]
A_52_P447944	NM_008532	5.04	8.96	Mus musculus tumor-associated calcium signal transducer 1 (Tacstd1), mRNA [NM_008532]
A_51_P418820	NM_009335	40.16	37.90	Mus musculus transcription factor AP-2, gamma (Tcfap2c), mRNA [NM_009335]
A_51_P338262	NM_011619	5.37	8.17	Mus musculus troponin T2, cardiac (Tnnt2), mRNA [NM_011619]
A_51_P482503	NM_009413	21.46	34.02	Mus musculus tumor protein D52-like 1 (Tpd52l1), mRNA [NM_009413]
A_51_P506822	NM_011674	42.90	81.82	Mus musculus UDP galactosyltransferase 8A (Ugt8a), mRNA [NM_011674]
A_52_P439263	NM_011674	56.91	70.50	Mus musculus UDP galactosyltransferase 8A (Ugt8a), mRNA [NM_011674]

**Genes expressed in common with the mouse orthologs of the human "intrinsic" gene list of published by Sorlie**				

A_51_P311038	NM_001024139	7.11	9.21	Mus musculus a disintegrin-like and metalloprotease (reprolysin type) with thrombospondin type 1 motif, 15 (Adamts15), mRNA [NM_001024139]
A_51_P366811	NM_007470	45.17	36.20	Mus musculus apolipoprotein D (Apod), mRNA [NM_007470]
A_51_P163015	NM_172309	2.77	14.02	Mus musculus aryl hydrocarbon receptor nuclear translocator-like 2 (Arntl2), mRNA [NM_172309]
A_51_P455647	NM_009801	7.57	9.21	Mus musculus carbonic anhydrase 2 (Car2), mRNA [NM_009801]
A_51_P302823	NM_019686	4.02	11.39	Mus musculus calcium and integrin binding family member 2 (Cib2), mRNA [NM_019686]
A_51_P165185	NM_016887	11.54	15.50	Mus musculus claudin 7 (Cldn7), mRNA [NM_016887]
A_52_P507877	NM_007729	41.32	30.23	Mus musculus procollagen, type XI, alpha 1 (Col11a1), mRNA [NM_007729]
A_51_P459477	NM_007729	34.69	28.45	Mus musculus procollagen, type XI, alpha 1 (Col11a1), mRNA [NM_007729]
A_51_P502614	NM_026268	4.28	6.46	Mus musculus dual specificity phosphatase 6 (Dusp6), mRNA [NM_026268]
A_51_P116940	NM_007940	15.92	5.95	Mus musculus epoxide hydrolase 2, cytoplasmic (Ephx2), mRNA [NM_007940]
A_51_P455932	NM_007974	12.81	33.87	Mus musculus coagulation factor II (thrombin) receptor-like 1 (F2rl1), mRNA [NM_007974]
A_52_P192418	NM_010175	2.63	4.90	Mus musculus Fas (TNFRSF6)-associated via death domain (Fadd), mRNA [NM_010175]
A_51_P382925	NM_010175	2.16	3.61	Mus musculus Fas (TNFRSF6)-associated via death domain (Fadd), mRNA [NM_010175]
A_52_P100252	NM_007988	3.23	3.40	Mus musculus clone:A630082H08 product:fatty acid synthase, full insert sequence. [AK080374]
A_52_P136162	NM_010206	2.27	4.37	Mus musculus fibroblast growth factor receptor 1 (Fgfr1), mRNA [NM_010206]
A_52_P668810	NM_010206		3.81	Mus musculus fibroblast growth factor receptor 1 (Fgfr1), mRNA [NM_010206]
A_51_P335000	NM_010211		5.05	Mus musculus four and a half LIM domains 1 (Fhl1), mRNA [NM_010211]
A_51_P329394	NM_008034	6.64	8.48	Mus musculus folate receptor 1 (adult) (Folr1), mRNA [NM_008034]
A_52_P217710	NM_008056		3.08	Mus musculus frizzled homolog 6 (Drosophila) (Fzd6), mRNA [NM_008056]
A_51_P282538	NM_008077	28.43	73.47	Mus musculus glutamic acid decarboxylase 1 (Gad1), mRNA [NM_008077]
A_52_P144310	NM_008077	27.33	68.46	Mus musculus glutamic acid decarboxylase 1 (Gad1), mRNA [NM_008077]
A_51_P380005	NM_015736	32.95	69.29	Mus musculus UDP-N-acetyl-alpha-D-galactosamine:polypeptide N-acetylgalactosaminyltransferase 3 (Galnt3), mRNA [NM_015736]
A_51_P379997	NM_015736	38.50	82.09	Mus musculus UDP-N-acetyl-alpha-D-galactosamine:polypeptide N-acetylgalactosaminyltransferase 3 (Galnt3), mRNA [NM_015736]
A_51_P462422	NM_008124		5.07	Mus musculus gap junction membrane channel protein beta 1 (Gjb1), mRNA [NM_008124]
A_52_P16419	NM_010271	2.43	6.66	Mus musculus glycerol-3-phosphate dehydrogenase 1 (soluble) (Gpd1), mRNA [NM_010271]
A_52_P415996	NM_008184		6.29	Mus musculus glutathione S-transferase, mu 6 (Gstm6), mRNA [NM_008184]
A_51_P179664	NM_008185	3.67	6.80	Mus musculus glutathione S-transferase, theta 1 (Gstt1), mRNA [NM_008185]
A_52_P281702	NM_010518	2.12	3.08	Mus musculus insulin-like growth factor binding protein 5 (Igfbp5), mRNA [NM_010518]
A_51_P204153	NM_010518	4.25	10.70	Mus musculus insulin-like growth factor binding protein 5 (Igfbp5), mRNA [NM_010518]
A_52_P401386	NM_010593	3.74	5.28	Mus musculus junction plakoglobin (Jup), mRNA [NM_010593]
A_52_P642488	NM_008430	24.28	26.37	Mus musculus potassium channel, subfamily K, member 1 (Kcnk1), mRNA [NM_008430]
A_51_P307964	NM_010662	3.11	6.39	Mus musculus keratin complex 1, acidic, gene 13 (Krt1-13), mRNA [NM_010662]
A_51_P324814	NM_010664	2.93	3.89	Mus musculus keratin complex 1, acidic, gene 18 (Krt1-18), mRNA [NM_010664]
A_52_P410685	NM_033073	12.42	14.59	Mus musculus keratin complex 2, basic, gene 7 (Krt2-7), mRNA [NM_033073]
A_51_P242400	NM_031170	2.56	3.75	Mus musculus keratin complex 2, basic, gene 8 (Krt2-8), mRNA [NM_031170]
A_51_P174943	NM_008485	6.90	10.66	Mus musculus laminin, gamma 2 (Lamc2), mRNA [NM_008485]
A_51_P220062	NM_008609	10.60	15.42	Mus musculus matrix metalloproteinase 15 (Mmp15), mRNA [NM_008609]
A_51_P514085	NM_013606	3.75	6.35	Mus musculus myxovirus (influenza virus) resistance 2 (Mx2), mRNA [NM_013606]
A_52_P608097	NM_008756	17.70	41.15	Mus musculus occludin (Ocln), mRNA [NM_008756]
A_51_P486150	NM_008756	10.05	12.72	Mus musculus occludin (Ocln), mRNA [NM_008756]
A_52_P458279	NM_011169	15.09	21.23	Mus musculus prolactin receptor (Prlr), mRNA [NM_011169]
A_51_P248345	NM_009334		17.92	Mus musculus transcription factor AP-2 beta (Tcfap2b), transcript variant 1, mRNA [NM_009334]
A_51_P418820	NM_009335	40.16	37.90	Mus musculus transcription factor AP-2, gamma (Tcfap2c), mRNA [NM_009335]
A_51_P240614	NM_008536	3.79	5.43	Mus musculus transmembrane 4 superfamily member 1 (Tm4sf1), mRNA [NM_008536]
A_51_P482503	NM_009413	21.46	34.02	Mus musculus tumor protein D52-like 1 (Tpd52l1), mRNA [NM_009413]
A_51_P106059	NM_009423	2.81	3.48	Mus musculus Tnf receptor associated factor 4 (Traf4), mRNA [NM_009423]
A_52_P78403	NM_009423	2.90	5.78	Mus musculus Tnf receptor associated factor 4 (Traf4), mRNA [NM_009423]

**Genes expressed in common with the MMTV/neu mammary tumor gene signature described by Landis**				

A_51_P320852	NM_007657	2.52	7.50	Mus musculus CD9 antigen (Cd9), mRNA [NM_007657]
A_51_P242265	NM_009950	2.45	4.36	Mus musculus CASP2 and RIPK1 domain containing adaptor with death domain (Cradd), mRNA [NM_009950]
A_52_P559779	NM_007883	17.30	22.26	Mus musculus desmoglein 2 (Dsg2), mRNA [NM_007883]
A_51_P249848	NM_007883	11.40	13.77	Mus musculus desmoglein 2 (Dsg2), mRNA [NM_007883]
A_52_P88091	NM_007883	22.00	38.20	Mus musculus desmoglein 2 (Dsg2), mRNA [NM_007883]
A_52_P601757	NM_007883	76.45	101.83	Mus musculus desmoglein 2, mRNA (cDNA clone IMAGE:4036406), partial cds [BC034056]
A_51_P502614	NM_026268	4.28	6.46	Mus musculus dual specificity phosphatase 6 (Dusp6), mRNA [NM_026268]
A_51_P246166	NM_007969	10.06	10.23	Mus musculus extracellular proteinase inhibitor (Expi), mRNA [NM_007969]
A_52_P329207	NM_007969	13.14	12.84	Mus musculus extracellular proteinase inhibitor (Expi), mRNA [NM_007969]
A_51_P329394	NM_008034	6.64	8.48	Mus musculus folate receptor 1 (adult) (Folr1), mRNA [NM_008034]
A_51_P352303	NM_011983	5.94	8.58	Mus musculus homer homolog 2 (Drosophila) (Homer2), mRNA [NM_011983]
A_52_P176573	NM_011983	7.52	12.27	Mus musculus homer homolog 2 (Drosophila) (Homer2), mRNA [NM_011983]
A_51_P510156	NM_008491	9.56	10.11	Mus musculus lipocalin 2 (Lcn2), mRNA [NM_008491]
A_52_P382785	NM_033525	3.71	12.17	Mus musculus nephronectin (Npnt), mRNA [NM_033525]
A_51_P289889	NM_033525	3.57	9.38	Mus musculus nephronectin (Npnt), mRNA [NM_033525]
A_52_P17556	NM_134249	6.84	18.37	Mus musculus T-cell immunoglobulin and mucin domain containing 2 (Timd2), mRNA [NM_134249]
A_51_P500906	NM_134249	12.27	24.24	Mus musculus T-cell immunoglobulin and mucin domain containing 2 (Timd2), mRNA [NM_134249]
A_51_P482503	NM_009413	21.46	34.02	Mus musculus tumor protein D52-like 1 (Tpd52l1), mRNA [NM_009413]

**Expression of genes associated with the human HER2/neu chromosome 17q amplicon by Bertucci**				

A_51_P317176	NM_009971	12.40	13.05	Mus musculus colony stimulating factor 3 (granulocyte) (Csf3), mRNA [NM_009971]
A_51_P216179	NM_010152	4.71	8.69	Mus musculus v-erb-b2 erythroblastic leukemia viral oncogene homolog 2, neuro/glioblastoma derived oncogene homolog (avian) (Erbb2), transcript variant 2, mRNA [NM_010152]
A_51_P230382	NM_008815	2.63	3.66	Mus musculus ets variant gene 4 (E1A enhancer binding protein, E1AF) (Etv4), mRNA [NM_008815]
A_51_P368823	NM_010346	3.09	4.64	Mus musculus growth factor receptor bound protein 7 (Grb7), mRNA [NM_010346]
A_52_P401386	NM_010593	3.74	5.28	Mus musculus junction plakoglobin (Jup), mRNA [NM_010593]
A_52_P567306	NM_010688	2.95	4.98	Mus musculus LIM and SH3 protein 1 (Lasp1), mRNA [NM_010688]
A_51_P182462	NM_010688	2.46	3.44	Mus musculus LIM and SH3 protein 1 (Lasp1), mRNA [NM_010688]
A_51_P387123	NM_011854	2.77	4.06	Mus musculus 2'-5' oligoadenylate synthetase-like 2 (Oasl2), mRNA [NM_011854]

^a^Average fold difference is the ratio (n = 3, p value cutoff > .05) MAM-1 or Bam1a RNA to Universal Mouse RNA. RNA from MAM-1 or Bam1a was labeled in red color (cy5), universal RNA is labeled in green (cy3). Three independent RNA preparations per condition were analyzed on three separate chips and averaged; a confidence cutoff of >0.95 was used for the raw values of red/green before normalization. The fold change (**MAM-1 or Bam1a/**Universal Mouse) = Normalized ratio value(cy5/cy3). The MAM-1 and Bam1a transcriptomes include genes that are differentially increased relative to the Universal mouse RNA by at least 2-fold.

Genes uniquely over-expressed by MAM-1 largely reflect the stromal signature of this breast cancer co-culture system. A select list of ~563 differentially expressed genes is provided [see Additional file 2]. We considered only differences greater than 3-fold to compensate for the dilution of tumor and stroma specific RNA in the MAM-1 cultures when compared to the cloned cell line, Bam1a. Certain genes that are uniquely over-expressed in Bam1a are likely to reflect the influence of co-culture on the gene-expression patterns. Paradoxically, for example, we observe >25-fold higher expression of EPSTI1 in Bam1a compared to MAM-1, which contradicts what is typically observed and expected [12]. A majority of the genes that are over-expressed in MAM-1 have been identified in tumor associated fibroblasts and stromal cells and represent genes involved in the fibrotic response and basement membrane synthesis [24,25]. In particular, collagen genes involved in fibrosis and contraction and growth factors that stimulate the fibrotic response. In addition, genes involved in remodeling the extracellular matrix, including ADAM and MMP family members are highly represented.

When we compared genes differentially expressed between Bam1a and MAM-1 to genes clusters used to determine the stromal signatures of breast cancers, we found that relative to Bam1a, MAM-1 over-expressed 70% of the genes associated with the desmoid-type fibromatosis signature described by West et al. [24] including *WISP2*, *COL1A1*, *COL5A1*, *COL3A1*, *COL6A1*, *MMP23*, *MMP19*, *CNN1*, *CTGF*, *ADAM19*, *FBN1 *and *ADAM12 *(Table 2). This cluster of stroma specific genes, also identifies subgroups of breast carcinomas with a more favorable outcome when compared to the solitary fibrous tumor cluster [24] Further analyses revealed similarities between MAM-1 and an invasion specific cluster that is associated with the desmoplastic response to invading breast cancer [25] (Table 2). In particular, we observe >20-fold over-expression of *COL1A1*, *IGFBP7 *and *SPARC *in MAM-1 which are correlated with panstromal and juctatumoral stromal cell responses involved in matrix remodeling and angiogenesis, respective. These data are consistent with the histological features of the primary lesion used to establish MAM-1 and demonstrate the preserved expression of invasion specific genes in the MAM-1 co-culture. We also observed upregulation of a variety of stroma specific genes associated with tumor progression, invasion and the malignant phenotype in MAM-1. These include, *MMP2 *[10,18]*ADAM12 *[26], *HAS2*, [27] and *CLIC4 *[28,29]. Others have also demonstrated that heterologous co-cultures of breast tumor cells with tumor-derived fibroblasts (but not normal skin fibroblasts) was required for myofibroblast differentiation (i.e., expression of alpha-SMA) and resembled the advanced stages of desmoplastic carcinomas [11], similar to what we observe in the homotypic MAM-1 co-culture model. In these studies, expression of *PMP22 *was identified as a candidate gene in modulating tumor cell interactions with fibroblast. In MAM-1, *PMP22 *is upregulated approximately 15-fold and thus, may also play a role in the MAM-1 model.

**Table 2:** Differentially expressed Fibromatosis and Desmoplastic response genes in the MAM-1 Transcriptome

**Agilent Accession**	**Genbank Accession**	**Ratio of MAM-1 vs. Bam1a**	**p-value (n = 3)**	**Gene Description and Symbol**	**Reference**
A_51_P162272	NM_009524	5.95	2.58E-03	Mus musculus wingless-related MMTV integration site 5A (Wnt5a), mRNA [NM_009524]	West
A_51_P390804	NM_016873	125.00	8.89E-06	Mus musculus WNT1 inducible signaling pathway protein 2 (Wisp2), mRNA [NM_016873]	West
A_51_P220343	NM_018865	16.66	4.06E-06	Mus musculus WNT1 inducible signaling pathway protein 1 (Wisp1), mRNA [NM_018865]	West
A_52_P658611	NM_007742	19.92	4.09E-03	Mus musculus procollagen, type I, alpha 1 (Col1a1), mRNA [NM_007742]	West, Iacobuzio-Donahue
A_52_P525107	NM_007742	187.97	1.12E-05	Mus musculus procollagen, type I, alpha 1 (Col1a1), mRNA [NM_007742]	West, Iacobuzio-Donahue
A_51_P377094	NM_007742	37.17	9.02E-08	Mus musculus procollagen, type I, alpha 1 (Col1a1), mRNA [NM_007742]	West, Iacobuzio-Donahue
A_51_P414637	NM_015734	168.63	7.15E-06	Mus musculus procollagen, type V, alpha 1 (Col5a1), mRNA [NM_015734]	West
A_52_P684242	NM_009930	4.93	7.45E-05	Mus musculus procollagen, type III, alpha 1 (Col3a1), mRNA [NM_009930]	West
A_51_P515605	NM_009930	94.34	1.67E-09	Mus musculus procollagen, type III, alpha 1 (Col3a1), mRNA [NM_009930]	West
A_51_P474496	NM_009933	12.21	6.30E-08	Mus musculus procollagen, type VI, alpha 1 (Col6a1), mRNA [NM_009933]	West
A_51_P502132	NM_011985	7.69	1.96E-07	Mus musculus matrix metalloproteinase 23 (Mmp23), mRNA [NM_011985]	West
A_51_P269166	NM_021412	13.33	1.11E-02	Mus musculus matrix metalloproteinase 19 (Mmp19), mRNA [NM_021412]	West
A_51_P293087	NM_008606	4.76	7.12E-06	Mus musculus matrix metalloproteinase 11 (Mmp11), mRNA [NM_008606]	West
A_51_P404077	NM_020510	3.45	3.09E-06	Mus musculus frizzled homolog 2 (Drosophila) (Fzd2), mRNA [NM_020510]	West
A_52_P597634	NM_021457	4.55	5.25E-07	Mus musculus frizzled homolog 1 (Drosophila) (Fzd1), mRNA [NM_021457]	West
A_52_P224801	NM_007993	130.03	1.47E-03	Mus musculus fibrillin 1 (Fbn1), mRNA [NM_007993]	West
A_51_P467224	NM_007993	240.38	8.77E-05	Mus musculus fibrillin 1 (Fbn1), mRNA [NM_007993]	West
A_52_P220176	NM_015814	20.88	3.70E-02	Mus musculus dickkopf homolog 3 (Xenopus laevis) (Dkk3), mRNA [NM_015814]	West
A_52_P489295	NM_009621	4.22	2.72E-05	Mus musculus a disintegrin-like and metalloprotease (reprolysin type) with thrombospondin type 1 motif, 1 (Adamts1), mRNA [NM_009621]	West
A_52_P213932	NM_009621	3.10	1.94E-05	Mus musculus a disintegrin-like and metalloprotease (reprolysin type) with thrombospondin type 1 motif, 1 (Adamts1), mRNA [NM_009621]	West
A_52_P280044	NM_009616	16.21	1.63E-05	Mus musculus a disintegrin and metalloproteinase domain 19 (meltrin beta) (Adam19), mRNA [NM_009616]	West
A_51_P267447	NM_009616	10.35	8.95E-04	Mus musculus a disintegrin and metalloproteinase domain 19 (meltrin beta) (Adam19), mRNA [NM_009616]	West
A_52_P290457	NM_007400	33.11	4.05E-03	Mus musculus a disintegrin and metalloproteinase domain 12 (meltrin alpha) (Adam12), mRNA [NM_007400]	West, Peduto
A_51_P510882	NM_007400	9.35	1.38E-03	Mus musculus a disintegrin and metalloproteinase domain 12 (meltrin alpha) (Adam12), mRNA [NM_007400]	West, Peduto
A_51_P350817	NM_009922	38.46	1.29E-04	Mus musculus calponin 1 (Cnn1), mRNA [NM_009922]	West
A_52_P599578	NM_145575	1.24	1.03E-02	Mus musculus caldesmon 1 (Cald1), mRNA [NM_145575]	West
A_51_P357573	NM_145575	1.25	3.07E-02	Mus musculus caldesmon 1 (Cald1), mRNA [NM_145575]	West
A_51_P157042	NM_010217	14.33	1.71E-04	Mus musculus connective tissue growth factor (Ctgf), mRNA [NM_010217]	West
A_51_P124748	NM_009368	0.45	7.08E-04	Mus musculus transforming growth factor, beta 3 (Tgfb3), mRNA [NM_009368]	West
A_51_P212754	NM_009369	11.76	1.54E-03	Mus musculus transforming growth factor, beta induced (Tgfbi), mRNA [NM_009369]	Iacobuzio-Donahue
A_51_P472292	NM_008048	65.79	6.80E-09	Mus musculus insulin-like growth factor binding protein 7 (Igfbp7), mRNA [NM_008048]	Iacobuzio-Donahue
A_51_P110301	NM_009778	4.59	3.95E-05	Mus musculus complement component 3 (C3), mRNA [NM_009778]	Iacobuzio-Donahue
A_51_P218774	NM_026418	2.74	9.21E-04	Mus musculus regulator of G-protein signalling 10 (Rgs10), mRNA [NM_026418]	Iacobuzio-Donahue
A_51_P224843	NM_021278	2.84	2.24E-03	Mus musculus thymosin, beta 4, X chromosome (Tmsb4x), mRNA [NM_021278]	Iacobuzio-Donahue
A_51_P111210	NM_133786	1.10	1.45E-01	Mus musculus SMC4 structural maintenance of chromosomes 4-like 1 (yeast) (Smc4l1), mRNA [NM_133786]	Iacobuzio-Donahue
A_52_P101852	NM_009242	27.25	1.90E-07	Mus musculus secreted acidic cysteine rich glycoprotein (Sparc), mRNA [NM_009242]	Iacobuzio-Donahue
A_51_P431087	NM_009242	67.11	1.96E-08	Mus musculus secreted acidic cysteine rich glycoprotein (Sparc), mRNA [NM_009242]	Iacobuzio-Donahue
A_51_P452876	NM_021515	7.46	3.48E-06	Mus musculus adenylate kinase 1 (Ak1), mRNA [NM_021515]	Iacobuzio-Donahue
A_51_P149562	NM_009686	6.13	1.52E-04	Mus musculus amyloid beta (A4) precursor protein-binding, family B, member 2 (Apbb2), mRNA [NM_009686]	Iacobuzio-Donahue
A_52_P49250	NM_010152	0.86	4.40E-03	Mus musculus v-erb-b2 erythroblastic leukemia viral oncogene homolog 2, neuro/glioblastoma derived oncogene homolog (avian) (Erbb2), transcript variant 2, mRNA [NM_010152]	Iacobuzio-Donahue
A_51_P216179	NM_010152	0.54	3.14E-03	Mus musculus v-erb-b2 erythroblastic leukemia viral oncogene homolog 2, neuro/glioblastoma derived oncogene homolog (avian) (Erbb2), transcript variant 2, mRNA [NM_010152]	Iacobuzio-Donahue
A_51_P341736	NM_008610	2.74	1.93E-04	Mus musculus matrix metalloproteinase 2 (Mmp2), mRNA [NM_008610]	Wang, Singer
A_51_P258529	NM_008885	15.77	3.73E-05	Mus musculus peripheral myelin protein (Pmp22), mRNA [NM_008885]	Kunz-Schughart
A_51_P213359	NM_008216	23.70	1.46E-07	Mus musculus hyaluronan synthase 2 (Has2), mRNA [NM_008216]	Li
A_52_P582374	NM_178825	0.04	5.59E-03	Mus musculus epithelial stromal interaction 1 (breast) (Epsti1), transcript variant b, mRNA [NM_178825]	Gudjonsson
A_51_P376050	NM_029495	0.03	2.78E-03	Mus musculus epithelial stromal interaction 1 (breast) (Epsti1), transcript variant a, mRNA [NM_029495]	Gudjonsson
A_51_P127681	NM_013885	3.62	1.28E-07	Mus musculus chloride intracellular channel 4 (mitochondrial) (Clic4), mRNA [NM_013885]	Suh, Ronnov-Jessen
A_51_P392687	NM_011701	25.000	5.47E-04	Mus musculus vimentin (Vim), mRNA [NM_011701]	
A_52_P56471	NM_007912	4.35	8.57E-06	Mus musculus epidermal growth factor receptor (Egfr), transcript variant 2, mRNA [NM_007912]	
A_51_P392242	NM_007912	4.17	1.10E-05	Mus musculus epidermal growth factor receptor (Egfr), transcript variant 2, mRNA [NM_007912]	
A_52_P674338	NM_207655	7.14	1.90E-03	Mus musculus epidermal growth factor receptor (Egfr), transcript variant 1, mRNA [NM_207655]	
A_52_P106259	NM_207655	11.56	2.06E-05	Mus musculus epidermal growth factor receptor (Egfr), transcript variant 1, mRNA [NM_207655]	
A_51_P414813	NM_010153	0.61	3.13E-03	Mus musculus v-erb-b2 erythroblastic leukemia viral oncogene homolog 3 (avian) (Erbb3), mRNA [NM_010153]	
A_51_P342050	NM_010153	0.58	3.28E-02	Mus musculus v-erb-b2 erythroblastic leukemia viral oncogene homolog 3 (avian) (Erbb3), mRNA [NM_010153]	
A_52_P420504	NM_007392	17.18	7.97E-05	Mus musculus actin, alpha 2, smooth muscle, aorta (Acta2), mRNA [NM_007392]	
A_52_P210078	NM_007392	101.94	6.74E-05	Mus musculus actin, alpha 2, smooth muscle, aorta (Acta2), mRNA [NM_007392]	
A_51_P103396	NM_016879	0.260	2.69E-04	Mus musculus keratin complex 2, basic, gene 18 (Krt2-18), mRNA [NM_016879]	
A_51_P356642	NM_008471	5.550	7.53E-06	Mus musculus keratin complex 1, acidic, gene 19 (Krt1-19), mRNA [NM_008471]	
A_51_P482128	NM_008469	2.760	3.01E-04	Mus musculus keratin complex 1, acidic, gene 15 (Krt1-15), mRNA [NM_008469]	
A_51_P118225	NM_028078	0.028	7.55E-04	Mus musculus junction adhesion molecule 4 (Jam4), mRNA [NM_028078]	
A_51_P358354	NM_023277	30.030	2.21E-06	Mus musculus junction adhesion molecule 3 (Jam3), mRNA [NM_023277]	
A_52_P374846	NM_023844	19.120	9.97E-04	Mus musculus junction adhesion molecule 2 (Jam2), mRNA [NM_023844]	
A_51_P418375	NM_023844	10.256	8.90E-04	Mus musculus junction adhesion molecule 2 (Jam2), mRNA [NM_023844]	
A_52_P93974	NM_021359	0.316	1.46E-02	Mus musculus integrin beta 6 (Itgb6), mRNA [NM_021359]	
A_52_P459521	NM_001005607	0.261	3.06E-05	Mus musculus integrin beta 4 (Itgb4), transcript variant 3, mRNA [NM_001005607]	
A_51_P382970	NM_133721	25.125	3.19E-02	Mus musculus integrin alpha 9 (Itga9), mRNA [NM_133721]	
A_52_P413034	NM_008398	2.985	2.73E-02	Mus musculus integrin alpha 7 (Itga7), mRNA [NM_008398]	
A_51_P181671	NM_008398	3.401	4.71E-03	Mus musculus integrin alpha 7 (Itga7), mRNA [NM_008398]	
A_52_P364140	NM_010577	30.030	1.76E-06	Mus musculus integrin alpha 5 (fibronectin receptor alpha) (Itga5), mRNA [NM_010577]	
A_52_P223495	NM_010576	0.304	4.71E-02	Mus musculus integrin alpha 4 (Itga4), mRNA [NM_010576]	
A_51_P515056	NM_013565	0.316	9.78E-05	Mus musculus integrin alpha 3 (Itga3), mRNA [NM_013565]	
A_51_P230405	NM_013565	0.260	5.62E-05	Mus musculus integrin alpha 3 (Itga3), mRNA [NM_013565]	
A_52_P612019	NM_008396	3.300	3.55E-02	Mus musculus integrin alpha 2 (Itga2), mRNA [NM_008396]	

^a^Average fold difference is the ratio (n = 3, p value cutoff > .05) compares the expression levels of MAM-1 RNA normalized to Universal Mouse RNA to Bam1a RNA normalized to Universal Mouse RNA. RNA from MAM-1 or Bam1a was labeled in red color (cy5), universal RNA is labeled in green (cy3). Three independent RNA preparations per condition were analyzed on three separate chips and averaged; a confidence cutoff of >0.95 was used for the raw values of red/green before normalization. The fold change (**MAM-1 or Bam1a/**Universal Mouse) = Normalized ratio value(cy5/cy3). P-values are for the comparisons between the MAM-1 and Bam1a. The references associated with specific genes are indicated in the text of the manuscript.

### Effect of Iressa on signal transduction in MAM-1

Western blot and microarray analyses revealed differential expression of the HER2/neu and EGFR in the respective tumor and stroma fractions of MAM-1 co-cultures. HER2/neu was exclusively expressed in the mammary tumor cell fraction and EGFR was approximately 8-fold higher in the stromal cell population. In so much as the EGFR is approximately 50-fold more sensitive to Iressa (IC_50_>0.080 μM) compared to the c-erbB-2 tyrosine kinase (IC_50_~3 μM) in vitro, we anticipated that EGFR in the stromal cell population was a likely target for the inhibitory activities of Iressa. Indeed, it has been suggested that targeting tumor associated fibroblasts [30] and the stromal EGFR may be a viable approach for preventing tumor progression and impeding the angiogenic switch. It has been shown that Iressa can differentially modulate fibrosis in various models of injury and disease [31-33]. It is important to distinguish, that in most injury models, the fibroblasts that are targeted as effectors of fibrosis are normal stromal fibroblasts and not phenotypically similar to the cancer associated fibroblasts/myofibroblasts that represent the stromal cells in the MAM-1 model. We expected that the response to Iressa in the MAM-1 stromal cells could be effected by their intrinsic sensitivity to Iressa and the microenvironment. Thus, we used the MAM-1 co-culture model to determine the selectivity and specificity of Iressa for the HER2/neu over-expressing mammary tumor cells versus the EGFR-expressing stromal cells by evaluating signal transduction downstream of these RTKs.

**Figure 5:**
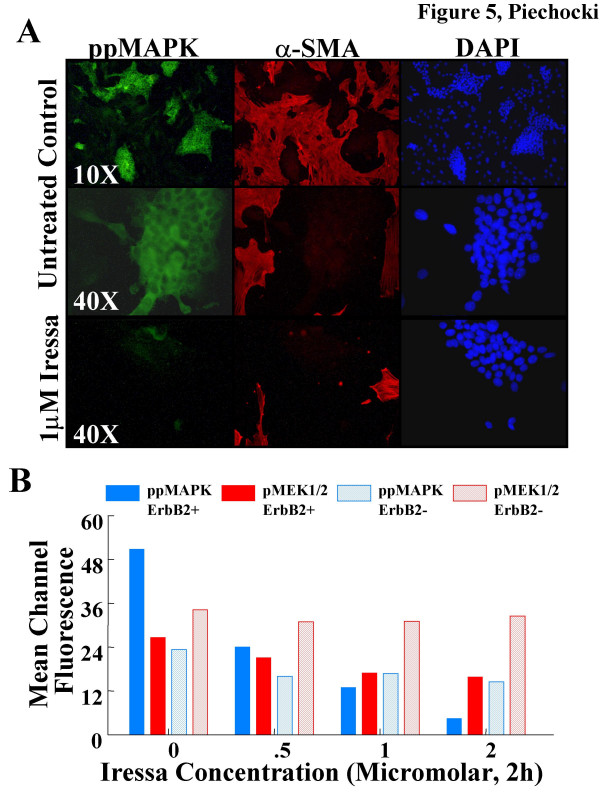
**Effect of Iressa on phosphorylation of p44/42 MAPK by (A) Immunofluorescence and phosphorylation of p44/42 MAPK and MEK1/2 by (B) flow cytometry in MAM-1 co-cultures**. MAM-1 were subcultured on coverslips or in 6-well plates and grown to ~95% confluence and then treated by replacing the conditioned media with fresh media that contained diluent (.001% DMSO, Control) or Iressa for 2 hours prior to fixing and evaluation as described in the methods. A. Immunofluorescent photomicrographs were taken with the indicated objectives of cells that were double labeled for phospho-p44/42 MAPK (Thr202/Tyr204) in green (FITC) and α-SMA in red (TRITC) and counterstained with DAPI (blue) to define the nuclei. B. Dose-response for phosphorylation of p44/42 MAPK and MEK1/2 in MAM-1 co-cultures by flow cytometry. MAM-1 were treated as described above and dual-labeled for ErbB-2 and the indicated phospho-specific antigen. Bars represent Mean channel fluorescent values for pp44/42 MAPK (Thr202/Tyr204) (Blue Bars) or pMEK1/2 (Ser217/221) (Red Bars) in ErbB2+ (Solid Bars) and ErbB2-(Shaded Bars) subpopulations.

In MAM-1 co-cultures that are stimulated by replacing the culture medium, strong cytoplasmic expression of phosphorylated p44/42 MAPK is readily observed (Fig. 5A). Inclusion of 1 μM Iressa eliminates this response. By flow cytometric analysis we determined the dose-response for p44/42 MAPK and pMEK1/2 in the ErbB-2+ (tumor cell) and ErbB-2-(stromal cell) subpopulations in MAM-1 co-cultures. We observed a dose-dependent decrease in pp44/42 MAPK and pMEK1/2 phosphorylation in tumor cells with maximal decreases of 90% and 40%, respective (Fig. 5B). We also observed a modest (38%) decrease in stromal cell phospho-pp44/42 MAPK at all doses of Iressa but no effect of pMEK1/2 phosphorylation, suggesting a small inhibitory effect of Iressa on the EGFR in the stroma. To determine the long term impact of these effects on cell growth and survival we treated MAM-1 co-cultures with Iressa for longer periods of time.

### Treatment of MAM-1 with Iressa generates a fibrotic response in vitro

**Figure 6:**
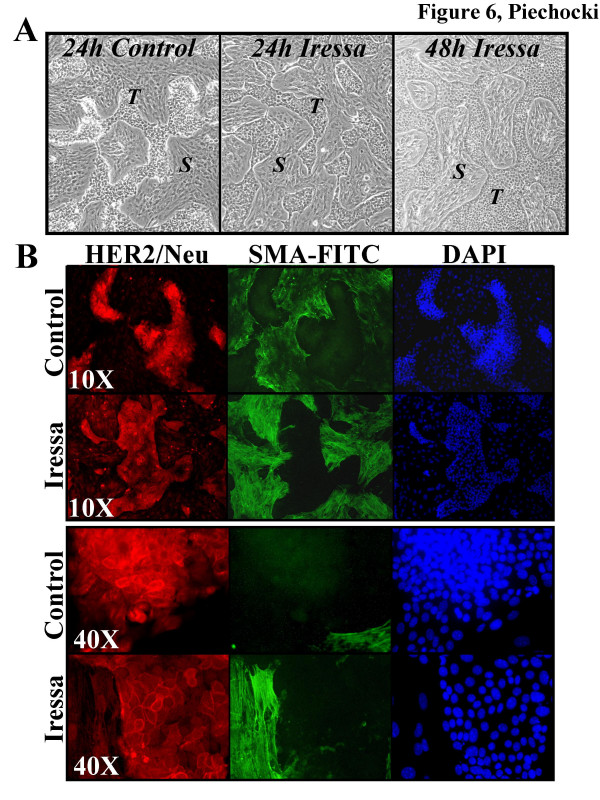
**Differential Effects of Iressa on growth and morphology**. MAM-1 co-cultures were treated for 24–48 h by replacing conditioned media with fresh media in the absence (Control) or presence of 1 μM Iressa. A. Phase contrast photomicrographs of MAM-1 co-cultures following treatment with 1 μM Iressa were taken using a 10× objective. Tumor cell nests (*T*) decrease in cellularity over time and stromal cells (*S*) thicken and increase in cellularity. By 48 h tumor cell nests erode, cells flatten, and show evidence of apoptosis while stromal cells develop into multi-cell layer nests recapitulating the morphology of a fibrotic response in vitro. B. Immunofluorescence of MAM-1 that were dual-labeled with HER2/neu-TRITC and α-SMA-FITC to identify the tumor (red) and stromal (green) subpopulations, respectively. Photographs taken under low power (10×) show decreased cellularity of HER2^+ ^nests (red) and increased cellularity and density in the α-SMA^+ ^stroma (green). These shifts in the cellularity of the separate subpopulations are emphasized by the nuclear counterstain with DAPI (blue). Under higher magnification (40×) HER2^+ ^nests of tumor cells (red) have decreased in cellularity and flattened into a monolayer while the cellularity and α-SMA reactivity has increased in the stromal elements surrounding the tumor cells generating the thickened appearance that is associated with fibrosis. Note in particular the membrane accentuation of HER2/neu in the tumor cells treated with Iressa and the dense bundles of a-SMA reactive fibers in Iressa treated stroma.

**Figure 7:**
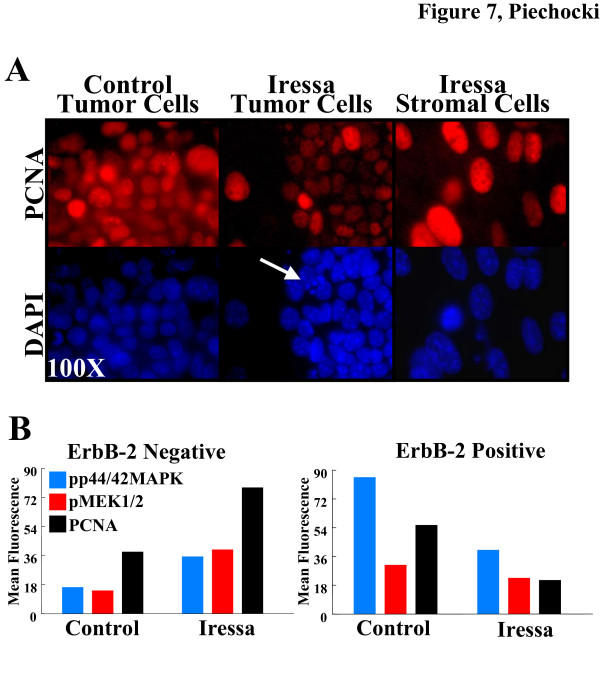
**Effect of Iressa on (A) PCNA activity and (B) Signal transduction in the tumor and stromal cell populations of MAM-1 co-cultures**. A. MAM-1 co-cultures were treated for 24 h with fresh media in the absence (Control) or presence of 1 μM Iressa then fixed and stained with PC10-TRITC to detect PCNA reactivity (red) and counterstained with DAPI to identify the nuclei (blue) Photomicrographs were taken with the 100× objective under oil immersion. The PCNA index of control tumor cells is >95% and <12% in the Iressa treated tumor cells. In the stroma adjacent to the Iressa treated tumor nest, PCNA index is >95%. Note the presence of apoptotic cells in DAPI stained tumor cells treated with Iressa (arrow). B. Differential effect of Iressa on PCNA, phospho-p44/42 MAPK (Thr202/Tyr204) and phospho-MEK1/2 (Ser217/221) in the ErbB-2 Negative (Stromal) and ErbB-2 Positive (Tumor) populations in MAM-1 treated for 24 h with 1 μM Iressa. Samples were dual-labeled for ErbB-2 and the indicated antigen and evaluated by dual-color flow cytometry. Bars represent the Mean Channel Fluorescent Values for phospho-p44/42 MAPK (Blue Bars), phospho-MEK1/2 (Red Bars) or PCNA (Black Bars). Following Iressa treatment, the ErbB-2 positive population decreased by 44% and the α-SMA positive population increased 3-fold in these MAM-1 co-cultures.

When treated for an extended period of time with Iressa, the morphology of the MAM-1 co-culture recapitulated a fibrotic response such that tumor cell nests and islands gradually eroded away and stromal cells increased in density forming multi-cell layer nests (Fig. 6A). Our primary observation was that the morphology and cellularity of the co-cultures was dramatically altered. Within 24 h of treatment with 1 μM Iressa, there was a decrease in the cellularity of tumor cell nests and an increase in the cellularity and density of α-SMA reactive material associated with the stromal cell layers (Fig. 6B). Decreased cellularity of the tumor cell nests is accompanied by significant tumor cell rounding and apoptosis as evidenced by nuclear fragmentation shown with DAPI staining (Fig 7A) as well as positivity for Annexin V binding and cleaved caspase 3 (not shown). In addition to apoptosis, when probed for PCNA, there was a marked reduction in tumor cell PCNA and robust staining of PCNA in the stromal cells (Fig. 7A). Basic flow cytometric evaluation of these cultures demonstrated a 44% reduction in the tumor cell population within 24 h of treatment with 1 μM Iressa and a >3-fold increase in the stromal cell population when compared to control cultures (*not shown*). When we evaluated the PCNA, phospho-p44/42 MAPK and phospho-MEK1/2 levels in the ErbB-2 positive and ErbB-2 negative subpopulations we observed a 62%, 54% and 27% reductions in tumor cell PCNA, phospho-p44/42 MAPK and phospho-MEK1/2, respective (Fig 7B). Interestingly, the larger subpopulation of tumor cells in these treated co-cultures were approximately 2-fold less responsive to Iressa in terms of PCNA and phospho-p44/42 MAPK levels attesting to the transient resistance afforded to cells likely in G2/M (not shown). In contrast to ErbB-2 positive tumor cells, ErbB-2 negative stromal cells had a robust increase in all of these proliferation markers following treatment with Iressa. PCNA levels increased by 205%, phospho-p44/42 MAPK increased 219% and phospho-pMEK1/2 increased by 279%. These data are in agreement with our qualitative observations that used immunofluorescence to document the relative density of the tumor and stroma cell populations in the MAM-1 co-cultures (Fig. 6B). The resilience of the stromal cell population is not limited to EGFR antagonists. We have also observed that while both the tumor and stroma cells express comparable levels of the TRAIL death receptor DR5, only the tumor cells are sensitive to TRAIL-mediated apoptosis (data not shown). Thus, targeting the stroma to effectively "sterilize" the microenvironment and inhibit tumor growth and progression presents a major challenge.

### Gene expression analysis reveals a signature of the fibrotic response

To generate a better understanding of the global response of MAM-1 to Iressa, we compared the gene expression profiles of MAM-1 co-cultures treated for 24 h with fresh media containing diluent or 1 μM Iressa. We observed a strong upregulation of inflammatory genes involved in the fibrotic response, consistent with the morphology of the treated MAM-1 co-cultures (Fig. 6A). Interestingly, none of the genes that were modulated by Iressa in Bam1a cells [6] were altered in MAM-1. We observed 2.5- to 6-fold increases in the expression of genes associated with inflammatory, fibrotic, pathological processes that are observed in a variety of diseases [34-37]. Most notably, we observed increases in inflammatory genes, (*C9*, *CCR7*, *TNFRS25*, *IL1F9*, *CCL22*, *TLR8*, *KLRA23*, *CLEC4N*, *CD22*), cell adhesion genes, (*COL4A3*, *SIGLEC5*, *LGALS12*, *CLDN5*) growth factor signaling and transcription genes, (*FGF22*, *CTGF*, *WNT8A*, *POU4F3*, *HEY1*) proliferation and differentiation (*CCNB3*, *MYB*, *TFF1*, *PROX1*) and proteolysis genes (*GZMK*, *TLL2*, *ADAM5*, *SERPINA1D*).

## Conclusion

Our data demonstrate the utility of the MAM-1 co-culture model in understanding the impact of the tumor microenvironment on the differential responses of invasive breast cancers and tumor associated myofibroblasts to chemotherapeutic agents and therapeutic modalities. Our data suggest that Iressa preferentially targets signal transduction from the tumor cell HER2/neu leading to tumor cell death and a fibro-proliferative response in the stroma. In this co-culture model tumor cells retain their intrinsic sensitivity to Iressa and the tumor associated myofibroblasts of the stroma demonstrate intrinsic resistance to Iressa. Understanding the tumor regulatory properties of this microenvironment before, during and after treatment will be critical in determining the most appropriate treatment modality to prevent progression and recurrence.

## Competing interests

The author declares that they have no competing interests.

## Pre-publication history

The pre-publication history for this paper can be accessed here:



## Supplementary Material

Additional file 1 MAM-1 Transcriptome. Data provides an annotated table listing >2000 genes elucidated by microarray analysis that are highly expressed (>2-fold relative to universal mouse RNA) in the MAM-1 model. Click here for file

Additional file 2 Differentially expressed genes between MAM-1 and Bam1a. Data provides an annotated table listing >560 genes elucidated by microarray analysis that are differentially expressed (>3-fold) between the cloned breast cancer cell line, Bam1a, and MAM-1 Click here for file
